# Exosome α-Synuclein Release in Plasma May be Associated With Postoperative Delirium in Hip Fracture Patients

**DOI:** 10.3389/fnagi.2020.00067

**Published:** 2020-03-13

**Authors:** Yi Yuan, Zhengqian Li, Ning Yang, Yongzheng Han, Xiaojuan Ji, Dengyang Han, Xiaoxiao Wang, Yue Li, Taotao Liu, Feng Yuan, Jindan He, Yajie Liu, Cheng Ni, Peng Zou, Geng Wang, Xiangyang Guo, Yang Zhou

**Affiliations:** ^1^Department of Anesthesiology, Peking University Third Hospital, Beijing, China; ^2^Department of Anesthesiology, Beijing Jishuitan Hospital, Beijing, China; ^3^Department of Cadre Health Care, Beijing Jishuitan Hospital, Beijing, China; ^4^Research Center of Clinical Epidemiology, Peking University Third Hospital, Beijing, China; ^5^Beijing National Laboratory for Molecular Sciences, Key Laboratory of Bioorganic Chemistry and Molecular Engineering of Ministry of Education, Synthetic and Functional Biomolecules Center, College of Chemistry and Molecular Engineering, Peking University, Beijing, China

**Keywords:** postoperative delirium, α-synuclein, exosome, hip fracture, geriatric (aging)

## Abstract

**Background**: Little is known about the underlying mechanisms of the similarities in the core features of postoperative delirium (POD) and α-synuclein (α-syn)-related cognitive disorders. We herein investigated associations between fluctuated levels of exosomal α-syn in the plasma and POD presentation in geriatric hip fracture patients.

**Methods**: We conducted an observational, prospective, and 1:1 matched (on age older than 65, hip fracture diagnosis, American Society of Anesthesiologist’ (ASA) physical status, duration of surgery, and intraoperative bleeding) case-control study: POD cases and non-POD controls were selected from the overall cohort by using Confusion Assessment Method (CAM). Delirium severity was measured by the Memorial Delirium Assessment Scale (MDAS). Plasma exosome levels of α-syn were examined preoperatively and at the time that POD was diagnosed, by using an established immunocapture technology based on a putative brain-cell-specific marker. Circulating concentrations of interleukin-1β (IL-1β), interleukin-6 (IL-6) and tumor necrosis factor-α (TNF-α) were also determined. The relationship between α-syn levels and POD risk, as well as the association between α-syn and MDAS scores and plasma cytokines, were assessed.

**Results**: POD incidence was 8.4% (17/202). Postoperative α-syn were either elevated or lowered. As primary outcome variables, the change of α-syn in POD patients was significantly higher than non-POD ones (21.0 ± 29.3 pg.ml^−1^ vs.1.9 ± 20.0, *P* = 0.047). The α-syn alteration was positively correlated to MDAS (*r* = 0.436, *P* = 0.010) and the change of IL-6 (*r* = 0.383, *P* = 0.025).

**Conclusions**: Exosome α-syn release in plasma may be associated with the POD development which might be due to systemic inflammation.

**Clinical Trial Registration**: www.clinicaltrials.gov, identifier ChiCTR-IPR-17012301.

**Prior Presentation**: The abstract of this work has been selected for presentation in the 2019 ANESTHESIOLOGY Journal Symposium “What’s New with the old,” and it has been present in the ASA 2019 annual meeting October 21st, 2019 in Florida.

## Introduction

Delirium is an acute and fluctuating brain dysfunction characterized by cognitive impairment and disturbance of consciousness. Postoperative delirium (POD) is commonly observed among older adult surgical patients during the postoperative period. It is associated with prolonged hospitalization, poor surgical outcomes, and greater healthcare costs (Whitlock et al., 2011). Despite the prevalence and clinical importance of POD, its pathophysiology is poorly understood, and no reliable biomarkers have been reported in previous studies.

α-synuclein (α-syn) is a neuronal protein of 140 residues that localizes predominantly to presynaptic terminals (Burre, 2015). There are several similarities in the core features of POD and α-syn-related cognitive disorders, including Parkinson’s disease (PD) dementia and dementia with Lewy bodies; these include fluctuating attention, visual hallucinations, and disorganized thoughts (Sunwoo et al., 2013; Gore et al., 2015; Vardy et al., 2015). A previous clinical study demonstrated that α-syn pathologies in the stomach are associated with POD after gastrectomy (Sunwoo et al., 2013). More recently, the same research group reported a significant correlation between POD and PD-related non-motor symptoms (Kim et al., 2018). Given that non-motor symptoms may represent the burden of α-syn deposit, POD is thus hypothesized to be a series of α-syn-related cognitive disorders, and maybe a preclinical stage of α-synucleinopathy (Sunwoo et al., 2013; Gore et al., 2015; Kim et al., 2018).

α-syn is abundant and self-propagates throughout the brain, and can also be transferred to peripheral tissues through exosomes, which are small membranous vesicles secreted by virtually all cell types (Danzer et al., 2012). Moreover, α-syn is not restricted to the brain, but can also be detected in peripheral tissues and in several body fluids, including plasma and saliva (Malek et al., 2014). In the plasma, α-syn can be found in its free form or in association with exosomes (Matsumoto et al., 2017). Exosomes carry disease-specific cargos and reflect changes that occur during several neurodegenerative diseases, and the involvement of exosomes in transmitting α-syn pathology has been clearly demonstrated (Vella et al., 2016; Wu et al., 2017). In a previous study, it is reported that plasma exosomal α-syn is likely brain-derived [i.e., it is L1 cell adhesion molecular (L1CAM)-positive] and is increased in PD compared with healthy controls (Shi et al., 2014). However, to the best of our knowledge, no previous study has investigated the association between plasma exosomal α-syn and POD.

Elderly patients with hip fractures are prone to developing delirium after surgery for their injury, with the prevalence of POD ranging between 38% and 61% (Yoon et al., 2017). POD occurs more often in an early period postoperatively with arbitrary time courses ranging from postoperative day 0–1–5–30 days (Safavynia et al., 2018). Both trauma and surgical stress may contribute to neuroinflammation and the resultant POD occurrence. The inflammation cascade may be implicated in α-syn pathology and synaptic disruption (Alam et al., 2018). Since the plasma exosomal α-syn level was highly correlated to the a-syn content within the neuron, we hypothesized that patients with greater change amount of exosomal a-syn levels in plasma from a preoperative non-delirium state to an occurrence of POD would likely to be vulnerable to developing POD, accompanied by a more severe systemic inflammation. Accordingly, the present study was undertaken with the primary aim of exploring the association of the change of plasma α-syn in L1CAM-carrying exosomes and POD occurrence, and secondarily, to determine whether aforementioned exosome a-syn changed parallel to circulating inflammatory mediators in older adult hip fracture surgery patients.

## Materials and Methods

### Ethical Considerations

A prospective observational, single-center, 1:1 matched case-control preliminary study was conducted in Beijing Jishuitan Hospital. The study was approved by the Beijing Jishuitan Hospital ethics committee (Institutional Review Board: JLKS201705-04; Registration number: ChiCTR-IPR-17012301). Written informed consent was obtained from each enrolled patient accompanied by at least a family member or proxy.

### Patients and Controls

We enrolled patients aged 65 or older who experienced POD after an emergency or planned hip fracture surgery, under either general or regional anesthesia, between September 2017 and February 2018. All the patients were admitted to the orthogeriatric unit. Exclusion criteria were: preoperative delirium, Parkinson disease, all-cause dementia (including PD related dementia, AD-related dementia, and Lewy’s body dementia) alcohol-related disorders, multiple trauma or multiple fractures, acute or chronic infectious diseases, anti-inflammatory drug treatment, a stroke in the prior 6 months or any other central nervous system (CNS) disease, severe deafness or vision problems, linguistic barriers, illiteracy, and/or communication difficulties, patients who received blood transfusion perioperatively, transferred to ICU postoperatively and refusal or unexpected discharge.

**Figure 1:**
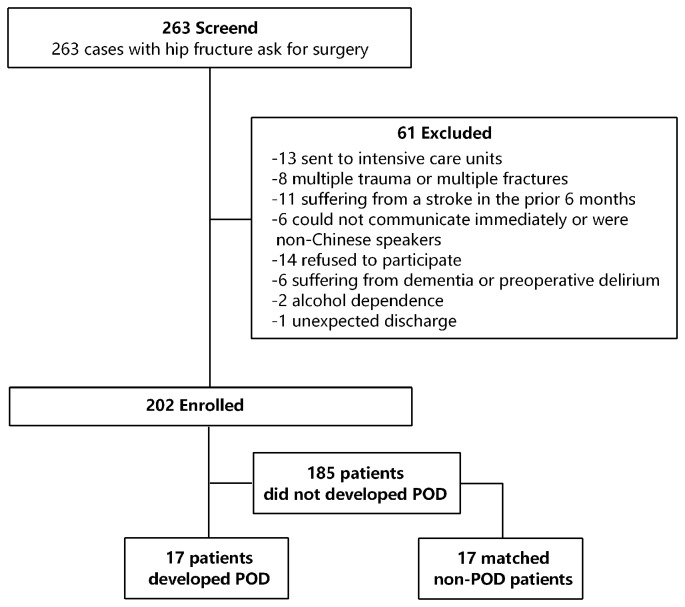
Flow diagram showed selection of eligible patients and the enrollment process. Abbreviations: POD, postoperative delirium.

POD cases and non-POD controls were frequency matched (1:1) on five variables using incidence density sampling. Specifically, one non-delirium control was randomly selected for each POD case from the source population according to the five matched variables, including age, diagnosis, American Society of Anesthesiologist’ (ASA) physical status, duration of surgery, and intraoperative blood loss. These variables shown in Supplementary Table S1 were listed in the European Society of Anesthesiology’s evidence- and consensus-based guidelines on POD and were considered to be risk factors for POD incidence after hip fracture (Aldecoa et al., 2017) surgery. A patient recruitment flow chart is shown in Figure 1.

We interviewed all patients the day before surgery and collected baseline data, including age, sex, gender, body mass index (BMI), ASA physical status, education history, and Mini-Mental State Examination (MMSE) score. Other information including comorbidities, past medical history, fracture classification, types of anesthesia and surgery, and time from injury to operation were also collected according to the patients’ medical records. All the history collection, physical evaluation and cognitive assessment related to dementia were conducted by the geriatrician (JXJ). Additionally, after cognitive assessment, written informed consent was signed with each patient accompanied by a family member or proxy.

### Anesthesia and Analgesia

All patients received ultrasound-guided fascia iliac block immediately after admission. For the block, a single injection of 30 mL of 0.33% ropivacaine was used. On the operating day, patients did not receive sedatives or anticholinergics before anesthesia. The eventual choice of anesthetic regimen (general anesthesia or neuraxial block) was based on the preference and experience of the anesthesiologist alongside discussion with the patient and their family. A uniform anesthetic regimen was conducted based on previous studies of orthopedic operations in older adults (Zhang et al., 2018). In order to minimize postoperative pain, a second fascia iliac block with the aforementioned protocol was provided right before anesthesia regardless of the anesthetic regimen. Furthermore, all patients received intravenous patient-controlled analgesia (PCA) postoperatively with the same analgesic regimen (sufentanil 100 μg and ondansetron 8 mg in 100 ml).

### Cognitive Scanner and Delirium Assessment

We interviewed all patients the day before surgery. The aforementioned MMSE test was used for dementia screening without any functional assessment. We followed the cut off by data from a Chinese cohort. Specifically, the cut-off points for dementia screening were 16/17 for illiterate (sensitivity 87.6% and specificity 80.8%), 19/20 for individuals with 1–6 years of education (sensitivity 93.6% and specificity 92.7%), and 23/24 for individuals with 7 or more years of education (sensitivity 94.3% and specificity 94.3%; Li et al., 2016).

The Confusion Assessment Method (CAM; a widely used, standardized method for the identification of delirium with a sensitivity of 94%, specificity of 89% and high interrater reliability) was used to exclude patients who had experienced preoperative delirium (Inouye et al., 2014). To be specific, four items (acute onset and fluctuating course, inattention, disorganized thinking, altered level of consciousness) were included in CAM, and if the first two were checked and at least one item of the last two was checked, a diagnosis of delirium was suggested. All participants were followed in the first two postoperative days, a period in which POD is usually diagnosed after hip fracture surgery in older adult patients (Scholtens et al., 2016). Surveillance included twice-daily visits (8:00 and 20:00) performed by a trained geriatrician (XJJ). POD patients were diagnosed using the CAM, and the severity of POD was determined by the Memorial Delirium Assessment Scale (MDAS) test (Marcantonio et al., 2002). Postoperative pain intensity was assessed using a 0–10-point Numerical rating scale (NRS; 0: no pain and 10: worst imaginable pain; Jensen et al., 1986).

### Blood Specimen Processing

Two venous blood samples (4 ml each) were collected from all the patients before the induction of anesthesia preoperatively. Postoperative blood samples (4 ml) were also collected either when delirium was diagnosed for POD cases or at the last visit on postoperative day 2 for non-POD controls. Blood samples were stored at 4°C immediately after collection and sent to centrifuge (at 3,000× *g* for 10 min) within 4 h to separate the plasma and blood cells. All the plasma samples were stored at −80°C before being sent to the Department of Pathology, Peking university third Hospital, for further processing.

### Exosome Isolation and α-syn Quantification

The primary outcome variable of this study was the change in exosomal α-syn concentration after a hip surgery. We evaluated plasma exosome levels of α-syn preoperatively and at the time that POD was diagnosed for cases or at a postoperative visit on day 2 for non-POD controls. Based on the preliminary study data and sample size estimation, we, therefore, chose the blood samples from 17 POD patients and 17 non-POD controls for further investigation with the method described in detail in the “Statistical Analysis” section and shown again in Figure 1.

Current strategies for purifying exosomes from blood or plasma differ significantly. There are basically three methods including ultracentrifugation-based isolation techniques, size-based isolation techniques, and immunoaffinity capture-based techniques. A recent study has demonstrated that immunoaffinity capture enriched for exosome and exosome-associated proteins by at least 2-fold more than the other two methods (Li et al., 2017). We, therefore, isolated the exosomes from plasma by using an immunoaffinity-based capture method as previously described (Li et al., 2017). As the founding member of subfamily of cell adhesion molecules, L1CAM was primarily expressed in the nervous system and was proved to be a high-affinity marker on the surface of exosomes derived from CNS (Shi et al., 2014). Therefore, anti-L1CAM antibodies were used for immunocapture of exosomes. Briefly, the method involves introducing anti-L1CAM antibodies to plasma to form an immunocomplex, binding the exosomes in the plasma to a solid phase through the immunocomplex, and then separating the solid phase and enriching the exosomes. Specific details refer as follows: 10 μg of L1CAM antibodies (clone UJ 127.11, Abcam, Cambridge, MA, USA) or normal mouse IgGs (Santa Cruz Biotechnology, Dallas, TX, USA) as negative controls were coated onto one set (1 mg) of M-270 Epoxy beads using a Dynabeads^®^ Antibody Coupling Kit (Life Technologies, Grand Island, NY, USA) according to the manufacturer’s instructions. After thawing quickly (within 2 min) at 37°C, plasma samples (>300 μl) were centrifuged at 2,000× *g* for 15 min followed by 12,000× *g* for 30 min, and then the supernatant was diluted 1:3 with phosphate buffered saline (PBS; pH 7.4). One set of antibody-coated beads and 900 μl of diluted plasma were incubated for ~24 h at 4°C with gentle rotation. The beads were then washed four times with 1 ml of 0.1% bovine serum albumin (BSA)/PBS (pH 7.4) and transferred into a new tube. Exosomes were eluted from the beads with 60 μl of a 1:1 mixture of 0.1% BSA/PBS (pH 7.4) and a fixing buffer (4% paraformaldehyde/5% glutaraldehyde) for electron microscopy imaging or lysed by incubating the beads in 110 μl of 1% Triton X-100 plus 10% of a protease inhibitor cocktail (P2714, Sigma-Aldrich; prepared in 10 ml of H_2_O) in 0.1% BSA/PBS (pH 7.4) for 1 h at room temperature with gentle shaking. The exosomal preparations were stored at −80°C until α-syn levels were measured using Luminex assays (Luminex, Austin, TX, USA) according to a previously published protocol (Hong et al., 2010).

### Determination of Plasma Cytokines

Concentrations of the plasma inflammatory cytokines interleukin-1β (IL-1β), interleukin-6 (IL-6), and TNF-α were determined in duplicate using a commercially available, sensitive enzyme-linked immunosorbent assay (#ab214025, #ab178013, and #ab181421, respectively; Abcam) according to the manufacturers’ instructions. All determinations were performed by laboratory technicians with no access to the clinical data.

### Statistical Analysis

The risk of POD was presented using odds ratios (OR) and 95% confidence intervals (CI) which was calculated by the Clopper-Pearson method. All selected risk factors and their associations with POD development were examined using univariate logistic regression analyses first. We also performed a multivariate analysis of factors related to POD onset by using a logistic regression model. Variables in the final model were selected according to a stepwise method and those deemed to have potential clinical importance (*P* < 0.2 in univariate logistic regression analyses) were included.

A power analysis was based on results from preliminary data comparing changes in plasma exosomal α-syn after surgery for hip fracture between groups. In our preliminary study, 5 of 50 patients experienced POD after surgery. Five non-POD controls were frequency-matched on age, diagnosis, ASA physical status, duration of surgery, and intraoperative blood loss. POD cases had greater increases in blood levels of α-syn in L1CAM-carrying exosomes from preoperative to postoperative compared with non-POD controls (Mean ± SD were 31.3 ± 13.8 pg.ml^−1^ and 15.8 ± 9.4 pg.ml^−1^, respectively). Sample-size calculations showed that a Student’s *t*-test with a type I error (two-sided) of *α* = 0.05 would have 95% power to detect the aforementioned difference in change of plasma exosomal α-syn between the two groups if the sample size in each group was 15. To account for a possible loss in the follow-up period, we enrolled 17 cases per group. All statistical analyses were performed using SPSS for Windows (version 14.0, SPSS, Chicago, IL, USA). Statistical significance was considered as *P* < 0.05.

## Results

### Participant Characteristics

A total of 263 consecutive patients underwent hip surgery from April 2017 to February 2018; of these, the majority were over the age of 65. Two-hundred and two older adult patients (age range: 65–89 years) were enrolled in our study. Of the enrolled patients, 17 subjects experienced POD during the first two postoperative days. The incidence of POD was 8.4%. Another 17 patients that did not experience POD over the first 2 postoperative days were also enrolled in this study (Figure 1).

The sample characteristics of the match variables stratified by POD cases and non-POD controls are illustrated in Supplementary Table S1. There were no significant differences between cases and controls for any of the five matched variables (age, diagnosis, ASA physical status, duration of surgery, and intraoperative blood loss), suggesting a successful matching procedure.

### Risk Factors of POD

Patient characteristics are shown in Table 1. Univariate logistic analysis of the potential risk factors for POD in older adult hip fracture patients demonstrated that there were no significant differences in a series of preoperative risk factors (all *P* > 0.05). However, univariate logistic analysis identified that a greater increase in exosomal α-syn from preoperative to postoperative was a risk factor for POD in older adult hip fracture patients (crude OR = 1.033, 95% CI 1.001–1.066, *P* = 0.047). Besides, it was also worth mentioning that the MDAS scores were significantly different between two groups, which not only confirmed that two groups were correctly classified by CAM but also added suitability of MDAS to be used for subsequent correlation analysis between MDAS scores and α-syn change. Furthermore, factors that remained statistically significant in the multivariate analysis are shown in (Table 2). Both the change in exosomal α-syn from preoperative to postoperative (adjusted OR = 1.044, 95% CI 1.003–1.087, *P* = 0.034) and preoperative MMSE (adjusted OR = 0.857, 95% CI 0.752–0.978, *P* = 0.022) were significantly different between the groups, which implied that patients with a higher increase in α-syn level and a lower preoperative MMSE score were more likely to develop POD.

**Table 1:** Univariate logistic analysis of potential factors for POD in geriatric hip fracture patients.

Variable	POD (*n* = 17)	Non-POD (*n* = 17)	Crude odds ratio (95% CI)	*P*-values
Age (year)	81.0 (6.0)	79.0 (6.0)	1.064 (0.948–1.195)	0.291
Gender (female/male)	8/9	12/5	0.370 (0.090–1.521)	0.168
Body mass index (kg.m^−2^)	24.1 (2.8)	23.7 (3.8)	1.043 (0.845, 1.288)	0.693
Education level (year)	6.0 (0.0, 19.0)	9.0 (0.0, 16.0)	1.037 (0.921, 1.167)	0.550
Diagnosis (femoral neck/Intertrochanteric fracture)	7/10	6/11	1.283 (0.321, 5.134)	0.724
ASA physical status (I/II/III)	1/11/5	1/11/5	1.000 (0.292, 3.429)	1.000
Preoperative albumin (g.L^−1^)	39.9 (4.0)	41.3 (3.5)	0.894 (0.726, 1.101)	0.293
Preoperative haemoglobin (g.L^−1^)	116.0 (15.0)	118.0 (12.0)	0.985 (0.935, 1.038)	0.571
Preoperative blood glucose (mmol.L^−1^)	7.8 (1.7)	8.8 (2.5)	0.797 (0.561, 1.131)	0.203
Preoperative serum sodium levels (normal/abnormal)	0/17	0/17	/	1.000
Preoperative MMSE scores	21.0 (10.0, 29.0)	24.0 (15, 30.0)	0.935 (0.857, 1.020)	0.129
Preoperative exosomal α-syn (pg.ml^−1^)	30.2 (19.0)	31.3 (28.6)	0.998 (0.970, 1.027)	0.885
Time from injury to operation (h)	107.0 (52.1)	95.9 (65.4)	1.003 (0.991, 1.016)	0.581
Type of anesthesia (general/spinal)	7/10	8/9	0.788 (0.203, 3.057)	0.730
Duration of anesthesia (min)	99.0 (23.0)	98.0 (15.0)	1.003 (0.968, 1.039)	0.872
Duration of surgery (min)	63.0 (23.0)	60.0 (16.0)	1.010 (0.974, 1.046)	0.600
Intraoperative blood loss (ml)	220.0 (50.0, 1,100.0)	190.0 (20.0, 420.0)	1.003 (0.998, 1.008)	0.192
Transfusion amount (ml)	1071.0 (298.0)	1,112.0 (276.0)	0.999 (0.997, 1.002)	0.669
MDAS scores	13.706 (5.010)	3.059 (2.772)	1.631 (1.215, 2.189)	0.001
Change of exosome α-syn (pg.ml^−1^)	21.0 (29.3)	1.9 (20.0)	1.033 (1.001. 1.066)	0.047

*The categorical variables were expressed as counts. Normal data are given as mean (SD), whereas non-normal data are expressed as median (IQR). The factors of postoperative delirium (POD) were assessed using univariate logistic regression analysis. Abbreviations: POD, postoperative delirium; MMSE, mini-mental state examination; CI, confidence interval; ASA, American Society of Anesthesiologists; MDAS, memorial delirium assessment scale; α-syn, α-synuclein*.

**Table 2:** Logistic regression multivariable analysis of factors of POD.

	B	SE	Adjusted odds ratio (95% CI)	*P*-Values
Gender (female/male)	1.736	1.097	5.674 (0.661, 48.708)	0.114
Preoperative MMSE scores	0.154	0.067	0.857 (0.752, 0.978)	0.022
Intraoperative blood loss (ml)	0.005	0.004	1.005 (0.998, 1.013)	0.175
Change of exosomal a-syn (pg.ml^−1^)	0.043	0.020	1.044 (1.003, 1.087)	0.034
Constant	0.655	1.361		0.630

*Abbreviations: POD, postoperative delirium; MMSE, mini-mental state examination; CI, confidence interval; α-syn, α-synuclein*.

For postoperative pain intensity (Supplementary Table S2), univariate logistic analysis revealed significant differences in pain at rest (crude OR = 2.499, 95% CI 1.164–5.367, *P* = 0.019) and movement-evoked pain (crude OR = 1.727, 95% CI 1.102–2.707, *P* = 0.017) on postoperative day 2, but not on postoperative day 1 (pain at rest: crude OR = 1.102, 95% CI 0.713–1.704; movement-evoked pain: crude OR = 1.181, 95% CI 0.846–1.649, *p* > 0.05). However, it is important to note that some patients experienced delirium on postoperative day 1; this means that POD occurred before postoperative pain assessment in these patients. The pain intensity score during the first 48 h after surgery should therefore not be crudely treated as a potential risk factor for POD during statistical analysis in this study. For this reason, we have presented these data separately in Supplementary Table S2.

### Plasma Concentrations of Exosomal α-syn

**Figure 2:**
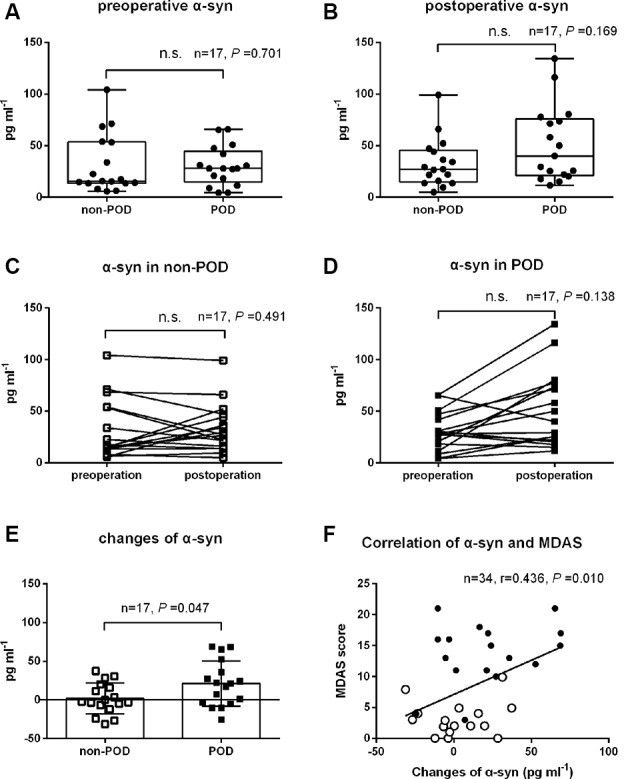
The changes in plasma exosomal α-syn concentration after hip fracture surgery in controls and POD patients. Data of preoperative and postoperative α-syn for controls and POD patients were nonnormally distributed and were described in Box-and-Whiskerr plots, presenting the minimum, maximum, and all points **(A,B)**. Before-after plots showing the trend of exosomal α-syn concentration before and after surgery in POD cases and non-POD controls **(C,D)**. The changes of plasma exosomal α-syn concentration from preoperative to postoperative (delirium detection for POD cases and a postoperative visit on day 2 for non-POD controls) were shown as mean and SD with all scatter points **(E)**. The changes of α-syn were positively correlated with MDAS scores (*r* = 0.436, *P* = 0.010), solid spots (black) represented POD cases and hollow spots (white) represented non-POD controls **(F)**. Abbreviations: POD, postoperative delirium; MDAS, memorial delirium assessment scale; α-syn, α-synuclein. n.s., non significant.

As shown in Figure 2; there were no significant differences in preoperative and postoperative plasma concentrations of exosomal α-syn between POD patients and controls (*P* = 0.70 and 0.17, respectively; Figures 2A,B). Similarly, there were no statistical differences between preoperative and postoperative plasma concentrations of exosomal α-syn in patients who experienced POD (*P* = 0.49) or in those who did not develop POD (*P* = 0.14; Figures 2C,D). However, univariate logistic analysis showed that the changes in exosomal α-syn concentration from preoperative to postoperative (delirium detection for POD cases and at a postoperative visit on day 2 for non-POD controls) were significantly higher in non-POD controls than in POD patients (1.9 ± 20.0 pg.ml^−1^ vs. 21.0 ± 29.3 pg.ml^−1^, *P* = 0.047; Figure 2E). By calculating the difference between the two groups (non-POD controls minus POD cases), the effect size is −19.1 pg.ml^−1^ (−36.7 pg.ml^−1^, −1.6 pg.ml^−1^). This implies that there is a greater alteration in plasma concentrations of exosomal α-syn in patients with POD. Furthermore, there was a positive correlation between the changes in plasma exosomal α-syn concentration and MDAS (a measure of delirium severity) scores (Figure 2F), however, this correlation was not significant in neither POD group nor non-POD one (data not shown).

### Plasma Concentration of Inflammatory Cytokines

**Figure 3:**
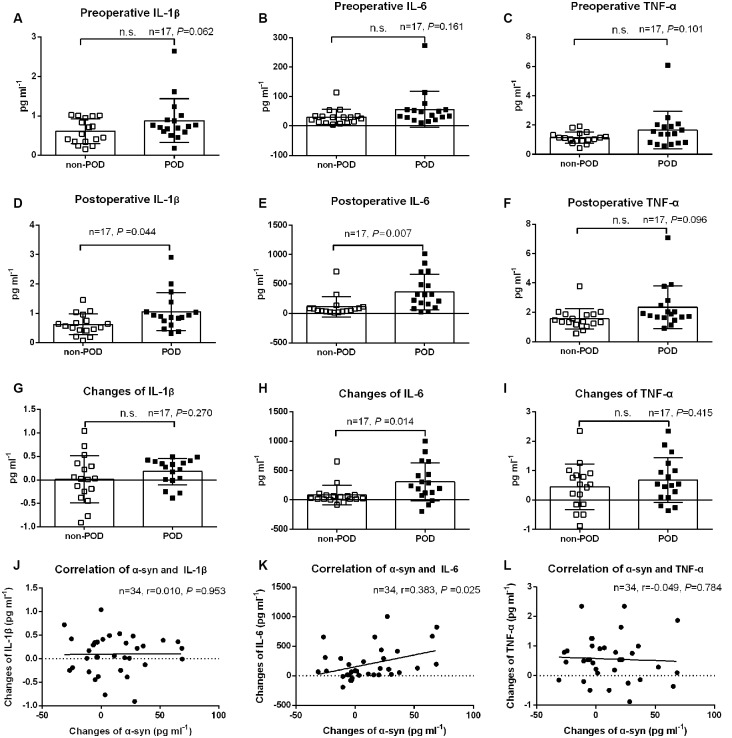
Plasma concentration of inflammatory cytokines. Data were shown as mean and SD with all scatter points. There were no significant difference in preoperative IL-1β, IL-6, and TNF-α between cases and controls **(A–C)**. Postoperative levels of IL-1β and IL-6, but, not TNF-α, were much higher in POD cases than those in non-POD controls **(D–F)**. The changes of plasma inflammatory cytokines from preoperative to the time that POD was diagnosed for cases or a postoperative visit on day 2 for non-POD controls **(G–I)**. Compared with non-POD patients, increases of plasma IL-6 concentration were much higher (*P* = 0.014); similarly, the changes of IL-6 (*r* = 0.383, *P* = 0.025), but not IL-1β and TNF-α, were positively correlated with postoperative increases in plasma exosomal α-syn **(J–L)**. Abbreviations: IL-1β, Interleukin-1β; IL-6, Interleukin-6; TNF-α, tumor necrosis factor-α; POD, postoperative delirium; α-syn, α-synuclein. n.s., non significant.

Neuroinflammation after surgery and anesthesia is thought to play a crucial role in POD development. There were no significant differences in preoperative IL-1β, IL-6, and tumor necrosis factor-α (TNF-α) between POD cases and non-POD controls (*P* > 0.05; Figures 3A–C). However, the postoperative levels of IL-1β and IL-6, but not TNF-α, were much higher in POD cases than in controls (*P* < 0.05; Figures 3D,E,F). We also compared the changes in concentration of the three cytokines from preoperative to postoperative, and there were no significant differences in IL-1β and TNF-α changes (*P* > 0.05; Figures 3G,I). However, the POD cases had a higher elevation in IL-6 concentrations than the non-POD controls (*P* < 0.05; Figure 3H). In addition, the relationship between changes in plasma exosomal α-syn and changes in inflammatory cytokines was examined using correlation analysis conducted with Prism 6. The changes in IL-6, but not in IL-1β or TNF-α, were positively correlated with changes in α-syn (*r* = 0.383, *P* = 0.025; Figures 3J–L).

## Discussion

Our results revealed for the first time that fluctuated (either increase or decrease) plasma exosomal α-syn level was an independent risk factor for POD. However, there is still no direct evidence that α-syn is involved in POD. An earlier study reported that α-syn pathologies and phosphorylation in the stomach are associated with POD after gastrectomy (Sunwoo et al., 2013), and this study group also reported a significant correlation between POD and PD-related non-motor symptoms, which may represent the burden of α-syn deposit (Kim et al., 2018). These indirect pieces of evidence strongly implied that POD represents a preclinical stage of α-syn-related cognitive disorders. In agreement with these, we observed a considerably higher alteration in plasma exosomal α-syn in POD cases when compared with non-POD controls. In addition, there was a positive correlation between the changes in plasma exosomal α-syn and MDAS score, which is a measure of delirium severity.

Many researches indicated that preoperative brain dysfunction (frail brain) of elderly patients is the predisposing factor for POD (Inouye et al., 2014). In our study, the multivariable analysis indicated that patients with lower preoperative MMSE were more vulnerable to POD (adjusted OR = 0.857, 95% CI 0.752–0.978, *P* = 0.022). Our finding is consistent with several previous studies that demonstrated that baseline cognitive deficits, even subtle ones, have been associated with an increased risk of developing delirium and the presence of dementia more than doubled the risk for POD (Maldonado, 2018). Therefore, it is essential to evaluate the baseline cognitive condition for elderly patients with hip fracture and communicate with the proxy the risk of delirium and the importance of early distinguish.

As one of the precipitating factors, inflammatory cytokines play a pivotal role during POD (Beloosesky et al., 2007; Whitlock et al., 2011; Liu et al., 2018). In the current study, both postoperative plasma concentration and elevation of IL-6 were significantly higher in POD cases than in non-POD controls. In contrast, the changes in IL-1β and TNF-α concentrations did not differ between POD cases and controls. Our results are consistent with a previously published meta-analysis, in which elevated peripheral levels of IL-6, but not IL-1β and TNF-α were detected in association with a POD (Liu et al., 2018). Although the exact mechanism of the correlation of a-syn change and delirium was not clear at present, the a-syn change might just be a bystander that confounded to the increase of IL-6, a marker of systemic inflammation. The detection of IL-6 may, therefore, be a good choice for inflammatory evaluation in future POD studies. We also found that the changes in peripheral IL-6 levels were positively correlated to the changes in α-syn, which demonstrated that there could be mutual regulation between neuroinflammation and α-synucleinopathy in the development of POD. Although few studies have investigated their association in POD, previous studies had shown their mutual association in neurodegeneration, including in PD and multiple system atrophy (Qin et al., 2016; Wong and Krainc, 2017; Gordon et al., 2018). The interesting finding of our study could be explained by this possible mechanism that it was the systemic inflammation that strong enough to cause POD to alter the dynamics of a-syn in the brain, that would be potentially initiated or exacerbate alpha synucleinopathies. Further investigations into the role of neuroinflammation in α-syn toxicity would thus be important for understanding α-syn pathophysiology in POD and may contribute to the development of future therapies to treat POD.

In our study, only 8.9% of patients experienced POD. This incidence was much lower than the overall incidence of POD of 23.3% (Yoon et al., 2017). We believe the most important reason lies in the orthogeriatric unit, a new model of care for our participants (González-Montalvo et al., 2010). The systematic organization of an orthogeriatric unit in our hospital allowed all participants to receive immediate regional nerve block analgesia, earlier geriatric assessment, coordinated daily clinical care, joint planning of the surgical schedule and discharge date and destination. Currently, this comprehensive intervention is thought to improve efficiency in perioperative management and reduce the incidence of delirium after hip fracture (González-Montalvo et al., 2010; Shields et al., 2017). Specifically, it is worth mentioning that the vast majority of participants in our study were discharged home or to a rehabilitation center on postoperative day 2, and delirium screening was then not available in our study. In fact, however, POD can occur up to 7 days after surgery. Furthermore, a number of previous studies have confirmed that pain is a risk factor for POD in older patients (Nie et al., 2012; Lin et al., 2016) and that effective pain management reduces the incidence and severity of POD (Lynch et al., 1998; Vaurio et al., 2006). We speculate the effective perioperative pain management, including twice preoperative fascia iliac blocks and postoperative intravenous PCA administration, may also partially account for the lower POD incidence in our study.

Our investigation had two limitations. First, two traumas, hip fracture, and surgery were in the patient population being studied, however, which one of them played an important role of the POD development remain largely unknown (Alam et al., 2018). Second, plasma total α-syn levels were not determined in our study. Although blood is more readily accessible, changes in α-syn concentrations in the blood of PD patients have been less consistent (Atik et al., 2016; Wang et al., 2018). Considering the abundance of α-syn in red blood cells and platelets that can substantially influence plasma α-syn levels, we, therefore, adopted a well-established immunocapture technology to isolate exosomes, and then examined brain-derived α-syn in the blood (Shi et al., 2014). Nevertheless, the patterns of change in plasma total and exosome-associated α-syn in POD patients need to be further investigated.

## Conclusions and Implications

In summary, our study indicates that fluctuated in plasma exosomal α-syn levels during the perioperative period might be associated with POD in older patients following hip fracture surgery. The change of α-syn level in L1CAM-carrying exosomes in the plasma was correlated with both IL-6 concentrations and POD severity, implying that an alteration in plasma exosomal α-syn may be implicated in the inflammatory process. Clinical determination of α-syn content in exosomes in plasma may, therefore, be helpful to differentiate older patients at risk of POD after hip fracture surgery.

## Data Availability Statement

All datasets generated for this study are included in the article/Supplementary Material.

## Ethics Statement

The studies involving human participants were reviewed and approved by Beijing Jishuitan Hospital ethics committee. The patients/participants provided their written informed consent to participate in this study.

## Author Contributions

YY, ZL and NY were the co-first authors of this article, responsible for the design and implementation of this project, data collection, data statistics, and article writing. XJ conducted neuropsychological tests. DH, YuL, TL and FY were responsible for exosome isolation and α-syn quantification. XW and CN participated in the analysis and interpretation of data. YY, YH, JH, and YaL were responsible for the patient data collection. YZ designed the project and modified the manuscript. XG was responsible for the expenses. PZ and GW also modified the manuscript. All authors read and approved the final manuscript.

## Conflict of Interest

The authors declare that the research was conducted in the absence of any commercial or financial relationships that could be construed as a potential conflict of interest.

## Abbreviations

α-syn: α-synuclein
ASA: American Society of Anesthesiologists’
BMI: Body Mass Index
BSA: Bovine Serum Albumin
CAM: Confusion Assessment Method
CI: Confidence Intervals
CNS: Central Nervous System
CSF: Cerebrospinal Fluid
IL-1β: Interleukin-1β
IL-6: Interleukin-6
IQR: Inter Quartile Range
L_1_CAM: L_1_-Cell Adhesion Molecular
MDAS: Memorial Delirium Assessment Scale
MMSE: Mini-Mental State Examination
NRS: Numerical Rating Scale
OR: Odds Ratios
PBS: Phosphate Buffered Saline
PCA: Patient Control Analgesia
PD: Parkinson’s disease
POD: Postoperative delirium
TICS: Telephone Interview for Cognitive Status
TNF-α: Tumor Necrosis Factor-α.
