# Supplementary material for: Exosome α-Synuclein Release in Plasma May be Associated With Postoperative Delirium in Hip Fracture Patients
Source: Front Aging Neurosci. 2020 Mar 13;12:67. doi: 10.3389/fnagi.2020.00067 (PMC7082759; doi:10.3389/fnagi.2020.00067)
Supplement: Supplementary file 1 [file Table_1.DOCX]

**Supplementary Table 1.** Frequency matching of five potential risk factors for POD in geriatric hip fracture patients.

| Matched factors | [Classification](javascript:;) | POD | Non-POD |
| --- | --- | --- | --- |
| Age (year) | <80 | 7 | 8 |
|  | ≥80 | 10 | 9 |
| Diagnosis | femoral neck fracture | 7 | 6 |
|  | Intertrochanteric fracture | 10 | 11 |
| ASA physical status | I | 1 | 1 |
|  | II | 11 | 11 |
|  | III | 5 | 5 |
| Duration of surgery (min) | ≤60 | 12 | 11 |
|  | ＞60 | 5 | 6 |
| Intraoperative blood loss (ml) | <300 | 11 | 13 |
|  | ≥300 | 6 | 4 |

Abbreviations: POD, postoperative delirium; ASA, American Society of Anesthesiologists.
