# Supplementary material for: Exosome α-Synuclein Release in Plasma May be Associated With Postoperative Delirium in Hip Fracture Patients
Source: Front Aging Neurosci. 2020 Mar 13;12:67. doi: 10.3389/fnagi.2020.00067 (PMC7082759; doi:10.3389/fnagi.2020.00067)
Supplement: Supplementary file 2 [file Table_2.DOCX]

**Supplementary Table 2.** The pain relief in POD patients versus non-POD patients.

| **Variable** | **POD (n=17)** | **Non-POD (n=17)** | **Crude odds ratio**  **(95% CI)** | ***P*-Values** |
| --- | --- | --- | --- | --- |
| VAS scores on postoperative day 1 |  |  |  |  |
| Pain at rest | 0.0 (0.0, 6.0) | 0.0 (0.0, 5.0) | 1.102 (0.713, 1.704) | 0.662 |
| Movement-evoked pain | 4.0 (1.0, 8.0) | 3.0 (0.0, 8.0) | 1.818 (0.846, 1.649) | 0.329 |
| VAS scores on postoperative day 2 |  |  |  |  |
| Pain at rest | 2.0 (0.0, 4.0) | 0.0 (0.0, 2.0) | 2.499 (1.164, 5.367) | 0.019 |
| Movement-evoked pain | 4.0 (0.0, 7.0) | 2.0 (0.0, 5.0) | 1.727 (1.102, 2.707) | 0.017 |

Non-normal data are expressed as median (IQR). Abbreviations: POD, postoperative delirium. VAS, visual analog scale.
